# Anaphylaxis to Banana and Persistent Sensitization to Thaumatin-Like Protein: An Emergent Culprit

**DOI:** 10.7759/cureus.79640

**Published:** 2025-02-25

**Authors:** Ana Luísa Pinhal, Ana Rodolfo, Borja Bartolomé, Alice Coimbra, Eunice Dias-Castro

**Affiliations:** 1 Allergy and Clinical Immunology Department, Unidade Local de Saúde de São João, Porto, PRT; 2 Research and Development Department, Roxall, Bilbao, ESP; 3 Public Health, Forensic Sciences, and Medical Education Department, Faculty of Medicine, University of Porto, Porto, PRT

**Keywords:** anaphylaxis, atopic march, banana allergy, sds-page immunoblotting, thaumatin-like proteins

## Abstract

Bananas contain different allergens, and reactions vary in severity. Thaumatin-like proteins (TLPs) present high stability during digestion and heat treatment. Data regarding reaction severity and acquisition of tolerance are lacking.

A two-year-old child presented with anaphylaxis after eating a banana. He was treated with corticosteroids and antihistamines on the way to the emergency department (ED) by his mother. Adrenaline was not administered as he had already improved upon arriving at the ED. The child had tolerated all fruits introduced into his diet, including bananas. He had never eaten kiwi. Skin prick tests (SPT) were positive for banana and kiwi and negative for aeroallergens, lipid transfer proteins, profilin, and latex. Skin prick-to-prick test (SPPT) with banana was positive. The specific IgE for bananas was 2.85 kUA/L. Sodium dodecyl sulfate-polyacrylamide gel electrophoresis (SDS-PAGE) immunoblotting with banana extracts and the patient’s serum showed an IgE-binding band compatible with Mus a 4 (banana TLP). Eviction of kiwis and bananas was recommended. At the age of six, the child presented with rhinoconjunctivitis during spring. SPT was positive for grass pollen and positive for banana and kiwi. The ImmunoCAP Immuno-Solid Phase Allergy Chip (ISAC®) test (Thermo Fisher Scientific, Uppsala, Sweden) demonstrated positive IgE against nAct d 2 (kiwi TLP), rPhl p 1, rPhl p 5, and rPhl p 11. Banana and kiwi eviction was maintained. Oral provocation was not performed. At the age of nine, the child started immunotherapy for grass pollen. Banana and kiwi SPPT remained positive.

This case illustrates the atopic march, with banana as the culprit food and TLP as the potential sensitizer. nAct d 2 is homologous to Mus a 4, which is not available in ISAC®. We highlight the severity of the reaction and the persistence of sensitization.

## Introduction

Fruit-induced anaphylaxis may occur in the context of pollen-food syndrome or independent of pollen allergy [1]. Information about the epidemiology, characteristics, and management of fruit allergy is sparse. Anaphylaxis may be defined as a "severe, life-threatening generalized or systemic hypersensitivity reaction" [2].

Allergic reactions to bananas (Musa acuminata) are uncommon worldwide, and they vary in severity, ranging from oral allergy syndrome to anaphylaxis [3]. Various banana allergens have been identified, such as Mus a 1 (profilin-actin binding protein), Mus a 2 (class 1 chitinase), Mus a 3 (nonspecific lipid transfer protein (nsLTP)), Mus a 4 (thaumatin-like protein), Mus a 5 (β-1,3-glucanase), and Mus a 6 (ascorbate peroxidase) [3].

Thaumatin-like proteins (TLPs) are a class of proteins that are structurally similar to thaumatin, a sweet-tasting protein identified in the plant Thaumatococcus daniellii [4]. These proteins belong to family five of pathogenesis-related proteins (PR-5). They have a molecular weight of 20-30 kDa and are resistant to proteases and exposure to heat or acid pH [5,6]. TLP has been described as an allergen in different fruits, pollens, and other plant-derived foods, such as apple (Mad d 2), peach (Pru p 2), kiwi (Act d 2), almonds (Pru du 2), pollens from olive (Ole e 13), cypress (Cup s 3), and cedar (Jun a 3 and Jun v 3) [1].

Data regarding the severity of reactions and the acquisition of tolerance related to TLP are lacking.

We report the case of a nine-year-old boy with a food allergy to bananas mediated by TLP.

## Case presentation

A child with a history of plagio-brachycephaly, corrected with helmet therapy, and atopic dermatitis, presented an anaphylactic reaction immediately after eating a banana at the age of two. He had oral discomfort, facial and tongue angioedema, emesis, rhinorrhea, and urticaria (legs). His mother administered dimethindene and betamethasone on the way to the emergency department, leading to a clinical improvement. At the hospital, hydroxyzine was given, as there was no need for adrenaline administration since the child had already improved. The boy remained under observation for 24 hours. On discharge, an adrenaline auto-injector, oral antihistamine, and oral corticosteroid were prescribed. The boy had eaten bananas before the reaction but always in small quantities because he refused, as he did not like bananas. He tolerated all the other fruits and vegetables introduced into his diet, but he had never eaten kiwi before.

Two months after the anaphylactic reaction, skin prick tests (SPT) with commercial extracts were positive for banana (4x3 mm) and kiwi (3x2 mm) and negative for D. pteronyssinus, grass pollen (Dactylis, Festuca, Lolium, Phleum, Poa), nsLTP (Pru p 3), pollen profilin, and latex. Other pollens were not tested due to the patient's age. Histamine (10 mg/ml) was 4x4 mm. Skin prick-to-prick tests (SPPT) with banana pulp and kiwi were positive. Total IgE was 11 kU/L, and basal tryptase was 3.48 microg/L; specific IgE to banana was 2.85 kUA/L and <0.35 kUA/L to latex.

**Figure 1:**
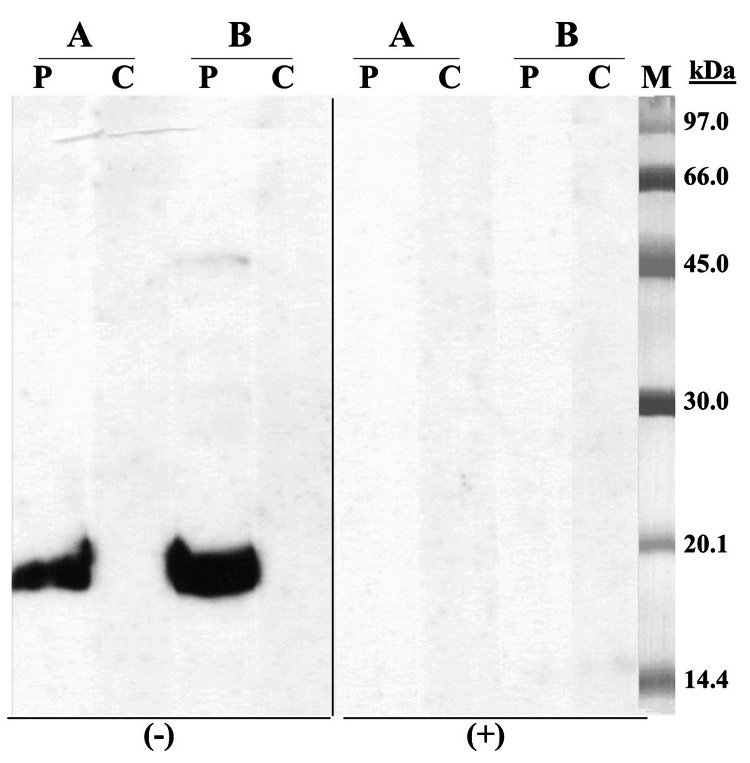
SDS-PAGE immunoblotting A: banana peel extract; B: banana pulp extract; Lane P: patient serum; Lane C: control serum (pool of sera from non-atopic subjects); Lane M: molecular mass standard. (-) samples without 2-mercaptoethanol, (+) samples with 2-mercaptoethanol. SDS-PAGE: sodium dodecyl sulfate-polyacrylamide gel electrophoresis

Protein extracts from banana peel and pulp were prepared by homogenization in phosphate buffer saline (20% W/V), dialyzation, and lyophilization. Sodium dodecyl sulfate-polyacrylamide gel electrophoresis (SDS-PAGE) immunoblotting in non-reducing conditions (without 2-mercaptoethanol) with banana peel and pulp extracts and the patient’s serum showed an IgE-binding band of approximately 19 kDa in both extracts; in reducing conditions (with 2-mercaptoethanol), no IgE-reactive band was detected in either of the banana extracts (Figure 1).

The loss of IgE-binding capacity by 2-mercaptoethanol treatment points to the presence of disulfide bonds in the structure of the IgE-reactive 19-kDa protein. At this point, the molecular mass of this protein and its IgE-binding capacity in the presence or absence of 2-mercaptoethanol (banana TLP has disulfide bonds stabilizing its three-dimensional structure) led us to consider Mus a 4 as the probable sensitizer. Oral provocation tests were not performed considering the severity of the initial reaction and the positive in vivo and in vitro tests.

Since the allergic reaction, the child has maintained avoidance of bananas and kiwis without any incidents.

At the age of six, the boy started showing ocular and nasal symptoms, namely sneezing, rhinorrhea, nasal obstruction, and ocular pruritis, suggestive of allergic rhinoconjunctivitis during the spring season. SPT with the following commercial extracts was performed: D. pteronyssinus, D. farinae, L. destructor, dog, cat, plane tree, birch, olive, cypress, grass pollen, weed pollen, Parietaria, Cladosporium herbarum, Aspergillus fumigatus, Alternaria, Blattella, banana, and kiwi. They were positive for grass pollen and remained positive for banana and kiwi extracts. The ImmunoCAP Immuno-Solid Phase Allergy Chip (ISAC®) test (Thermo Fisher Scientific, Uppsala, Sweden) demonstrated a positive specific IgE against nAct d 2 (5.1 ISAC standardized units for IgE (ISU-E))-kiwi TLP, rPhl p 1 (1.3 ISU-E), rPhl p 5 (0.8 ISU-E), and rPhl p 11 (0.4 ISU-E). The remaining allergens evaluated in ImmunoCAP ISAC® were negative, including Amb a 1. SPPT to kiwi and banana were repeated, and they were positive. Continued avoidance of banana and kiwi eviction was recommended.

At the age of nine, the child started immunotherapy for grass pollen. SPPT with kiwi and banana was performed once again with positive results. No other allergic reactions to fruits or vegetables have occurred until the present moment.

The main lab and skin test results of the patient are presented in Table 1.

**Table 1:** Lab findings and most relevant skin prick test results SPPT: skin prick-to-prick test; NA: not applicable

Lab or skin test (year of performance)	Result	Normal range
SPPT banana (2016)	Positive	NA
SPPT kiwi (2016)	Positive	NA
Total IgE (2016)	11kU/L	<107 kUA/L
sIgE banana (2016)	2.85 kUA/L	<0.35 kUA/L
sIgE latex (2016)	0.29 kUA/L	<0.35 kUA/L
Basal tryptase (2016)	3.48 µg/L	<11.40 µg/L
SPPT banana (2019)	Positive	NA
SPPT kiwi (2019)	Positive	NA
SPPT banana (2023)	Positive	NA
SPPT kiwi (2023)	Positive	NA

## Discussion

Kiwi TLP, Act d 2, presents a 74% sequence identity with Mus a 4, and they share common peptidic IgE epitopes [7]. Mus a 4 is not present in the ImmunoCAP ISAC® microarray. As the patient has never eaten kiwi fruit, cross-reactivity between banana and kiwi TLP surely explains the positive in vivo and in vitro allergy diagnostic results obtained with kiwi extract. Positive specific IgE to Mus a 4 (> 0.35 kUA/L) was detected in 72% of 51 sera from pediatric patients with banana allergy [7].

In the literature, we found another case report of a 22-year-old male with a banana allergy and TLP sensitization. He had a history of anaphylactic reactions to bananas and persimmons, and specific IgE to Act d 2 was detected in the ImmunoCAP ISAC® test. In this case, banana was the probable sensitizer, and the patient was advised to avoid banana, persimmon, and kiwi [8].

## Conclusions

The present case illustrates the atopic march, with the child presenting with atopic dermatitis in the first two years of life, followed by food allergy at two years of age, and then allergic rhinoconjunctivitis at the age of six.

In this particular child, TLP sensitization seems to be persistent over time and responsible for a severe allergic reaction. More studies are needed to understand the natural course of TLP allergy and the appropriate management of these patients. 
